# Penfluridol suppresses glioblastoma tumor growth by Akt-mediated inhibition of GLI1

**DOI:** 10.18632/oncotarget.16515

**Published:** 2017-03-23

**Authors:** Alok Ranjan, Sanjay K. Srivastava

**Affiliations:** ^1^ Department of Biomedical Sciences and Cancer Biology Center, Texas Tech University Health Sciences Center, Amarillo, TX 79106, USA; ^2^ Department of Immunotherapeutics and Biotechnology, Texas Tech University Health Sciences Center, Abilene, TX 79601, USA

**Keywords:** antipsychotic drug, glioblastoma, neurosphere, in vivo, intracranial

## Abstract

Glioblastoma (GBM) is the most common brain tumor with poor survival rate. Our results show that penfluridol, an antipsychotic drug significantly reduced the survival of ten adult and pediatric glioblastoma cell lines with IC_50_ ranging 2–5 μM after 72 hours of treatment and induced apoptosis. Penfluridol treatment suppressed the phosphorylation of Akt at Ser473 and reduced the expression of GLI1, OCT4, Nanog and Sox2 in several glioblastoma cell lines in a concentration-dependent manner. Inhibiting Akt with LY294002 and siRNA, or inhibiting GLI1 using GANT61, cyclopamine, siRNA and CRISPR/Cas9 resulted in enhanced cell growth suppressive effects of penfluridol. On the other hand, overexpression of GLI1 significantly attenuated the effects of penfluridol. Our results further demonstrated that penfluridol treatment inhibited the growth of U87MG tumors by 65% and 72% in subcutaneous and intracranial *in vivo* glioblastoma tumor models respectively. Immunohistochemical and western blot analysis of tumors revealed reduced pAkt (Ser 473), GLI1, OCT4 and increase in caspase-3 cleavage and TUNEL staining, confirming *in vitro* findings. Taken together, our results indicate that overall glioblastoma tumor growth suppression by penfluridol was associated with Akt-mediated inhibition of GLI1.

## INTRODUCTION

Glioblastoma (GBM) is one of the highly malignant and incurable brain tumors. The median survival rate of glioblastoma patients is only 12%. Despite availability of several therapeutic alternatives, glioblastoma still claims thousands of lives each year. Three major obstacles which exist in current treatment strategies for glioblastoma are (a) recurrence of tumor within six months after surgical removal (b) development of resistance to chemotherapies, and (c) inability of the drugs to cross blood-brain-barrier (BBB) in order to reach the brain. Due to lack of effective treatment, 88% of glioblastoma patients succumb to death within three years after diagnosis. Currently, glioblastoma is like a death sentence to the patients. Hence, it is critical to find new treatment options against this deadly disease.

Despite several advances, little is known about oncogenic signaling in glioblastoma. Hedgehog signaling pathway plays a critical role in glioblastoma tumor progression and pathogenesis [1]. GLI1, a transcription factor, belonging to sonic hedgehog pathway is over expressed in glioblastoma tumors [2]. GLI1 has also been shown to exhibit cancer stem cell property by overexpressing stem cell markers like OCT4, Nanog and Sox2. It has also been established that resistance of glioblastoma tumors to current therapies are related to overexpression of GLI1 [3]. Recently, PI3K/Akt mediated non-canonical activation of GLI1 has also been shown in renal cell carcinoma [4].

Published studies have shown reduced risk of cancer incidence in patients taking neuroleptic drugs [5]. Interestingly, thioridazine, an antipsychotic drug has been reported to inhibit glioblastoma tumor growth by inducing autophagy [6]. However, thioridazine is associated with severe cardiac toxicity thus preventing its clinical use [7]. Penfluridol is an oral antipsychotic drug used for schizophrenia. We have recently demonstrated that oral administration of penfluridol significantly suppresses the growth of metastatic breast tumors in brain, giving us the rationale to evaluate it against highly aggressive glioblastoma [8].

In the current study, we observed that penfluridol treatment significantly suppressed the growth of ten different glioblastoma cell lines. Our results showed Akt-mediated suppression of GLI1 with penfluridol treatment in various glioblastoma cell lines. Oral administration of penfluridol significantly suppressed the growth of subcutaneous and intracranial glioblastoma tumors through mechanism similar to what we observed in the *in vitro* experiments. To the best of our knowledge, this is the first study reporting suppression of glioblastoma tumor growth by penfluridol through Akt-mediated inhibition of GLI1.

## RESULTS

### Penfluridol suppresses the proliferation of patient derived adult and pediatric glioblastoma cell lines

**Figure 1:**
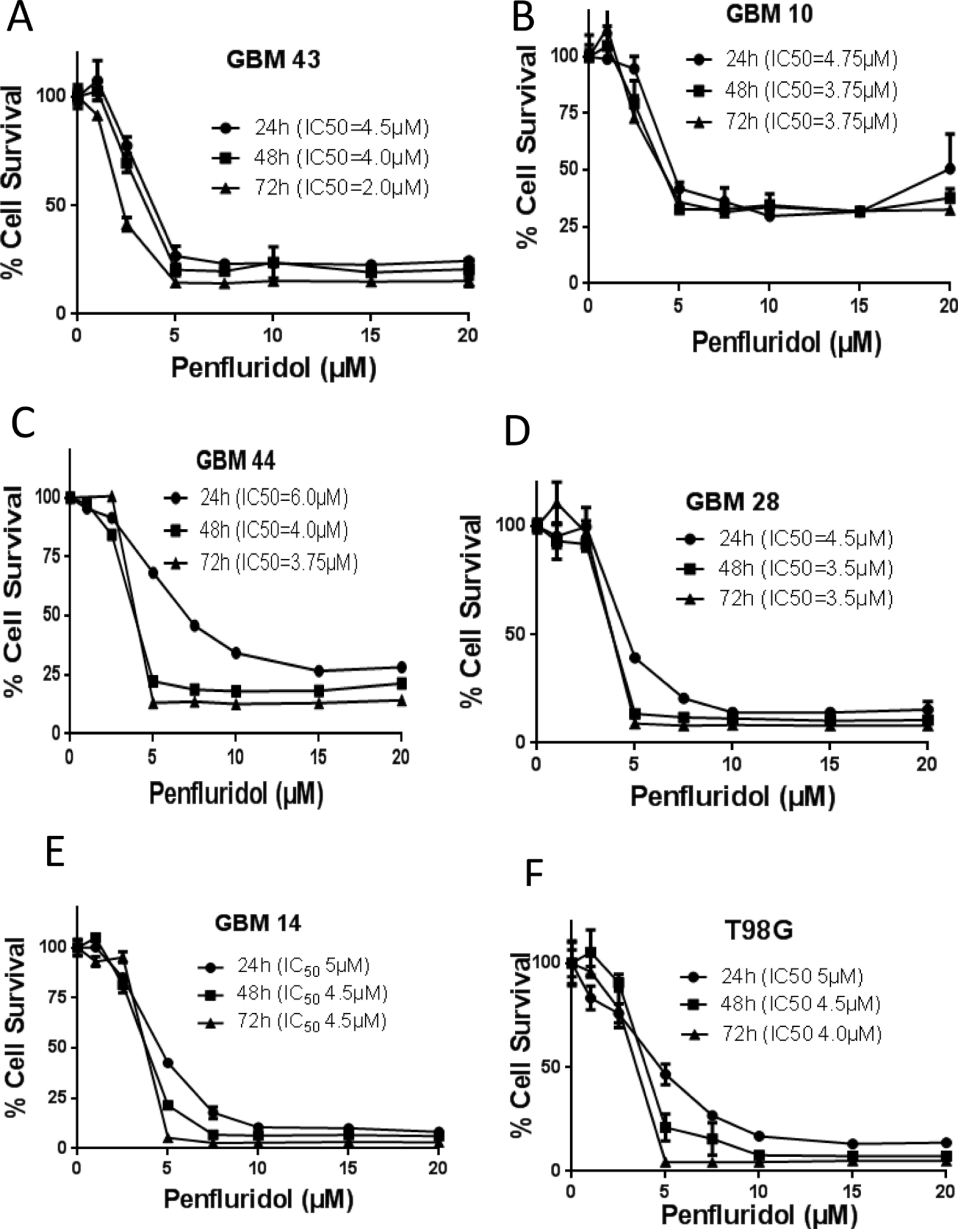

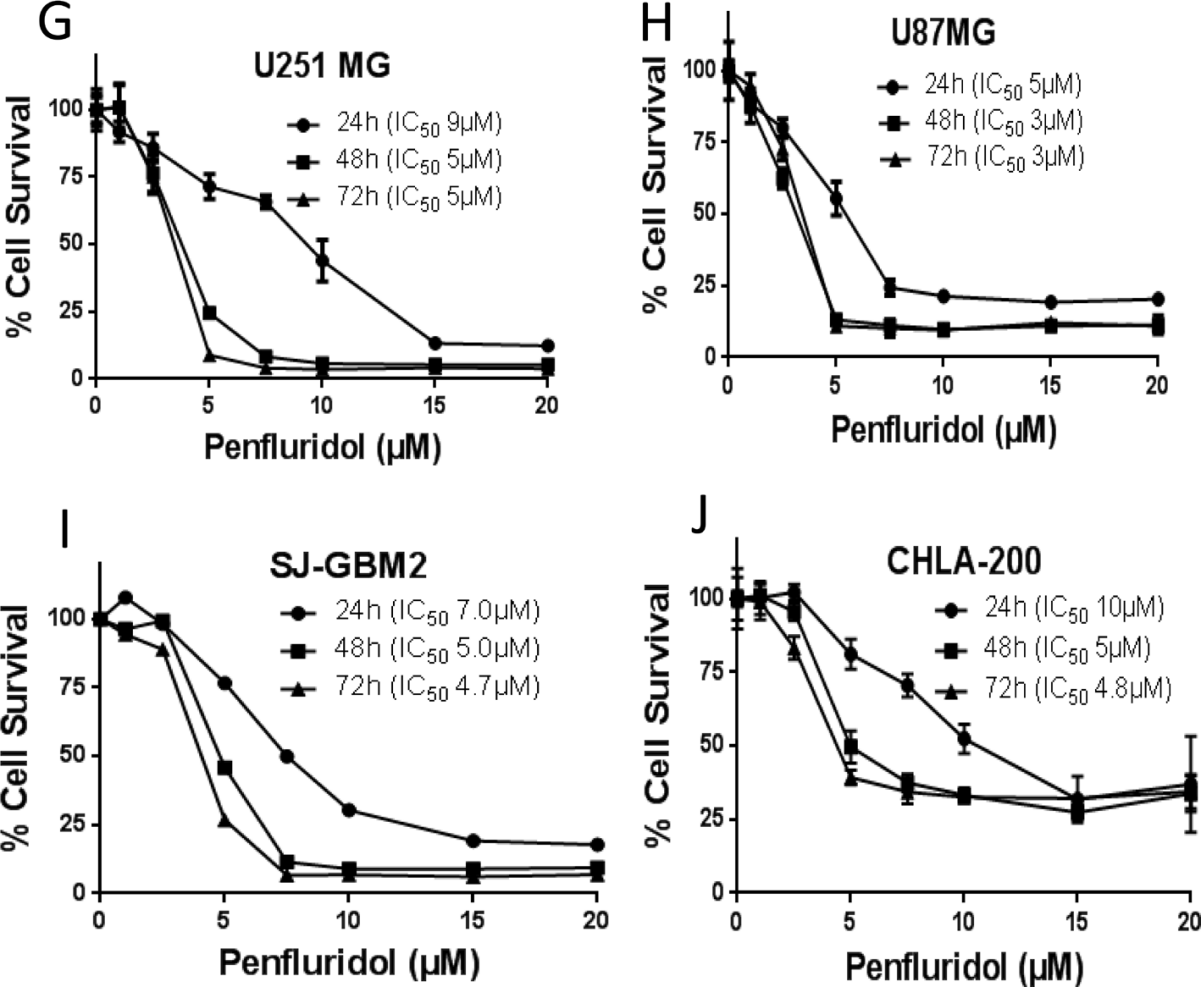
Penfluridol suppresses survival of glioblastoma cells (**A**) GBM43 (**B**) GBM10 (**C**) GBM44 (**D**) GBM28 (**E**) GBM14 (**F**) T98G (**G**) U251MG (**H**) U87MG (**I**) SJ-GBM2 and (**J**) CHLA-200 glioblastoma cells were treated with different concentrations of penfluridol for 24, 48 and 72 hours. Cell survival was measured by sulforhodamine B assay to estimate IC_50_ values. The experiments were repeated three times with 4–8 replicates in each experiment.

To evaluate the growth suppressive effects of penfluridol, we first performed the cytotoxicity assay in a panel of ten cell lines consisting of five adult patient derived (GBM43, GBM10, GBM44, GBM28, and GBM14), two pediatric patient derived (SJ-GBM2 and CHLA-200) as well as three established glioblastoma cell lines (U87MG, U251MG and T98G). Our results showed that with increasing concentrations, penfluridol significantly suppressed the growth of all the ten glioblastoma cell lines in a concentration and time-dependent manner. The IC_50_ of penfluridol after 24 h treatment ranged 4–10 μM in all the glioblastoma cell lines tested (Figure 1A–1J). The IC_50_ was reduced to about 2–5 μM after 48 and 72 h treatment (Figure 1A–1J). These results suggest the potential cytotoxic effects of penfluridol in glioblastoma cells.

### Induction of apoptosis by penfluridol

**Figure 2:**
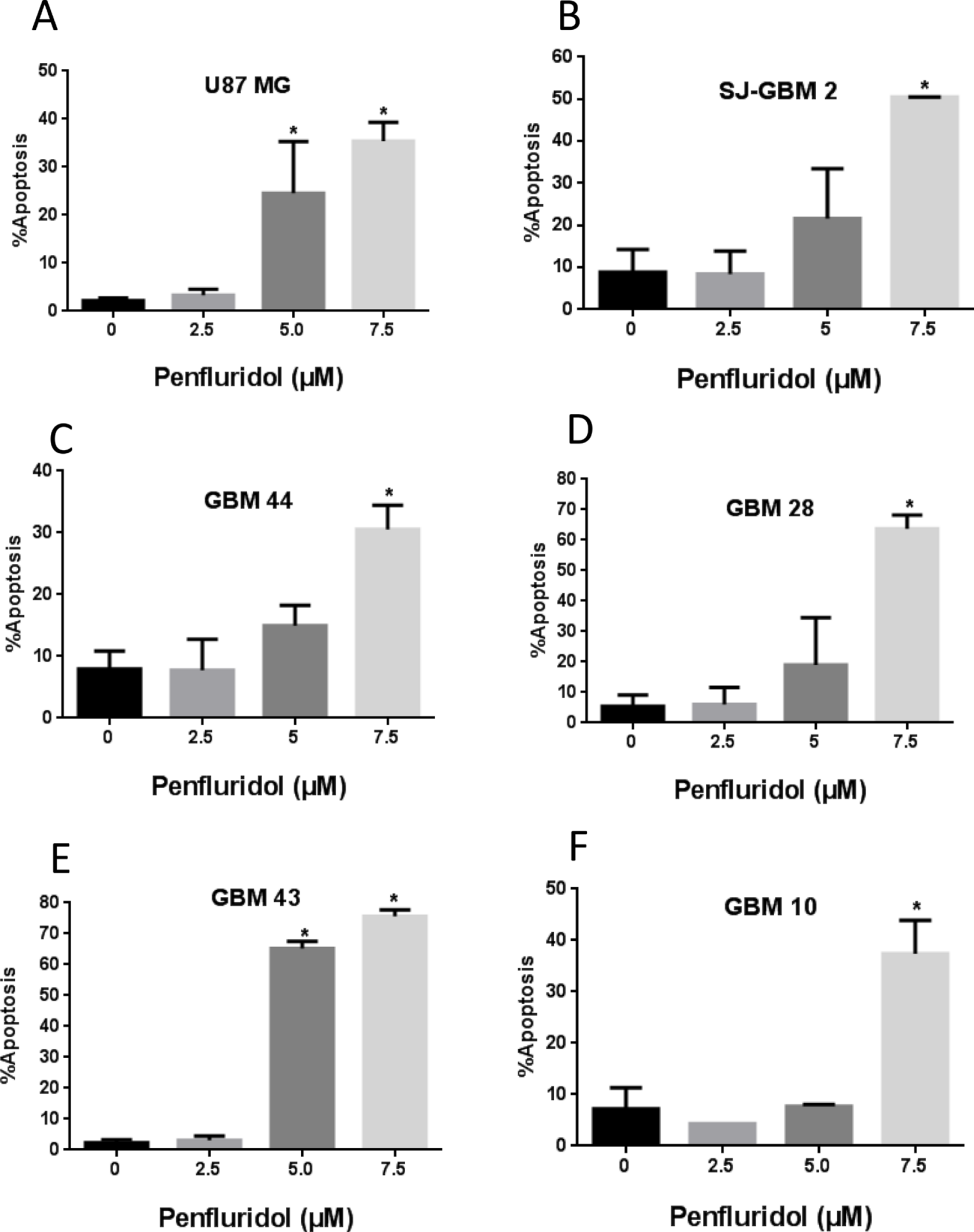
Penfluridol induce apoptosis in glioblastoma cells (**A**–**F**) Approximately 0.2–0.3 × 10^6^ U87MG, SJ-GBM2, GBM44, GBM28, GBM43 and GBM10 cells were plated in 6 well plates, treated with 2.5, 5.0 and 7.5 μM penfluridol for 48 h and processed for AnnexinV/FITC apoptosis assay using Accuri C6 flow cytometer. Values were plotted as means ± SD. Experiment was repeated three times. *Statistically significant at *p* < 0.05 when compared with control.

To determine the mechanism of growth suppressive effects of penfluridol, induction of apoptosis was evaluated in glioblastoma cells by AnnexinV/FITC assay using flow cytometer. As shown in Figure 2A–2FA, 48 h treatment with penfluridol resulted in significantly increased apoptosis in glioblastoma cells. The percent of apoptosis ranged 30–75% after 48 h of treatment with 7.5 μM penfluridol (Figure 2A–2F).

### Inhibition of Akt and sonic hedgehog signaling by penfluridol

**Figure 3:**
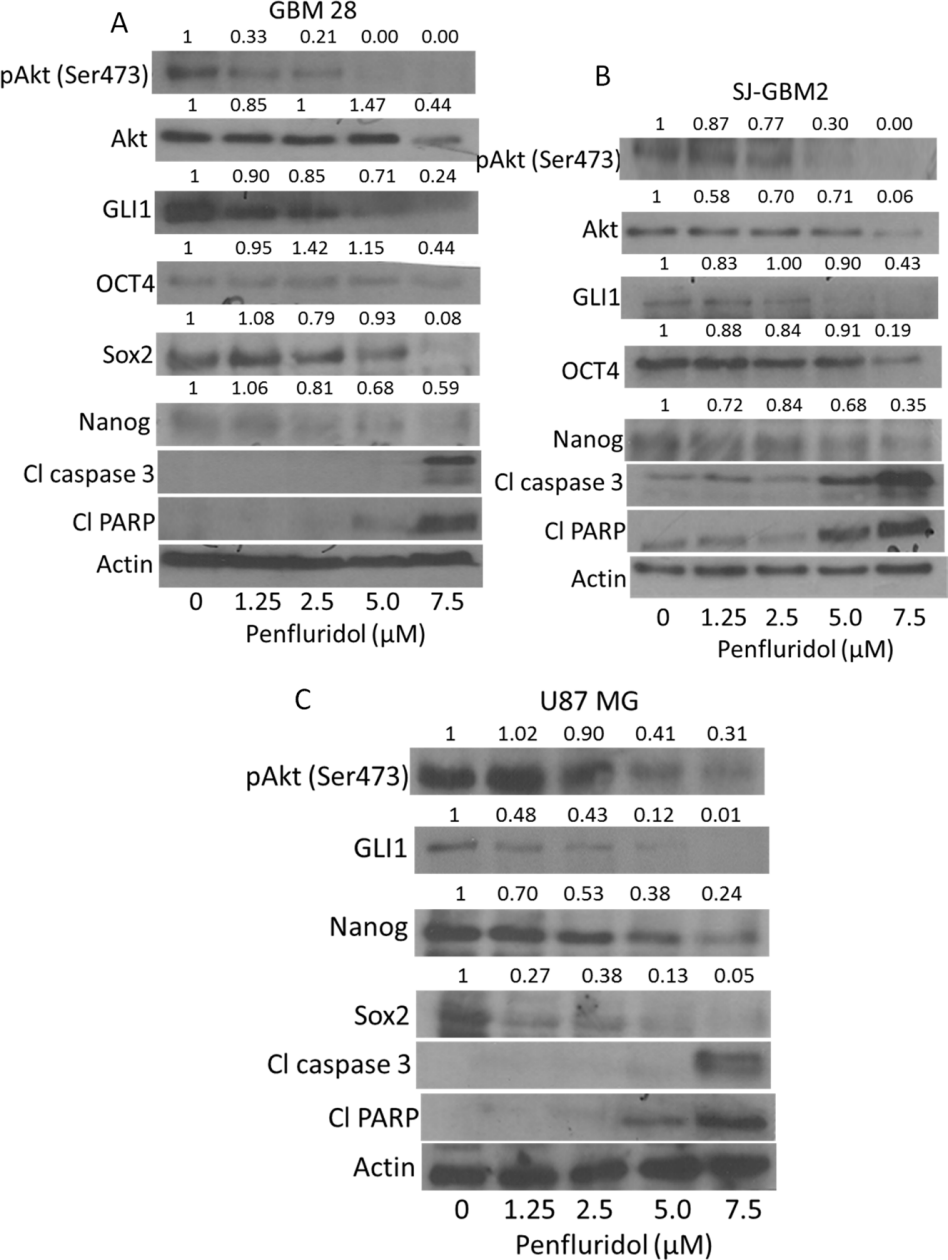
Penfluridol inhibits Akt-GLI1 signaling (**A**) GBM28, (**B**) SJ-GBM2, (**C**) U87MG cells were treated with different concentrations of penfluridol for 48 h. Representative blots showing concentration-dependent effect of penfluridol on pAkt(Ser473), Akt, GLI1, OCT4, Nanog, Sox2, Cl caspase 3 and Cl PARP. Actin was used as loading control. Figures shown are the representative blot of at least three independent experiments.

**Figure 4:**
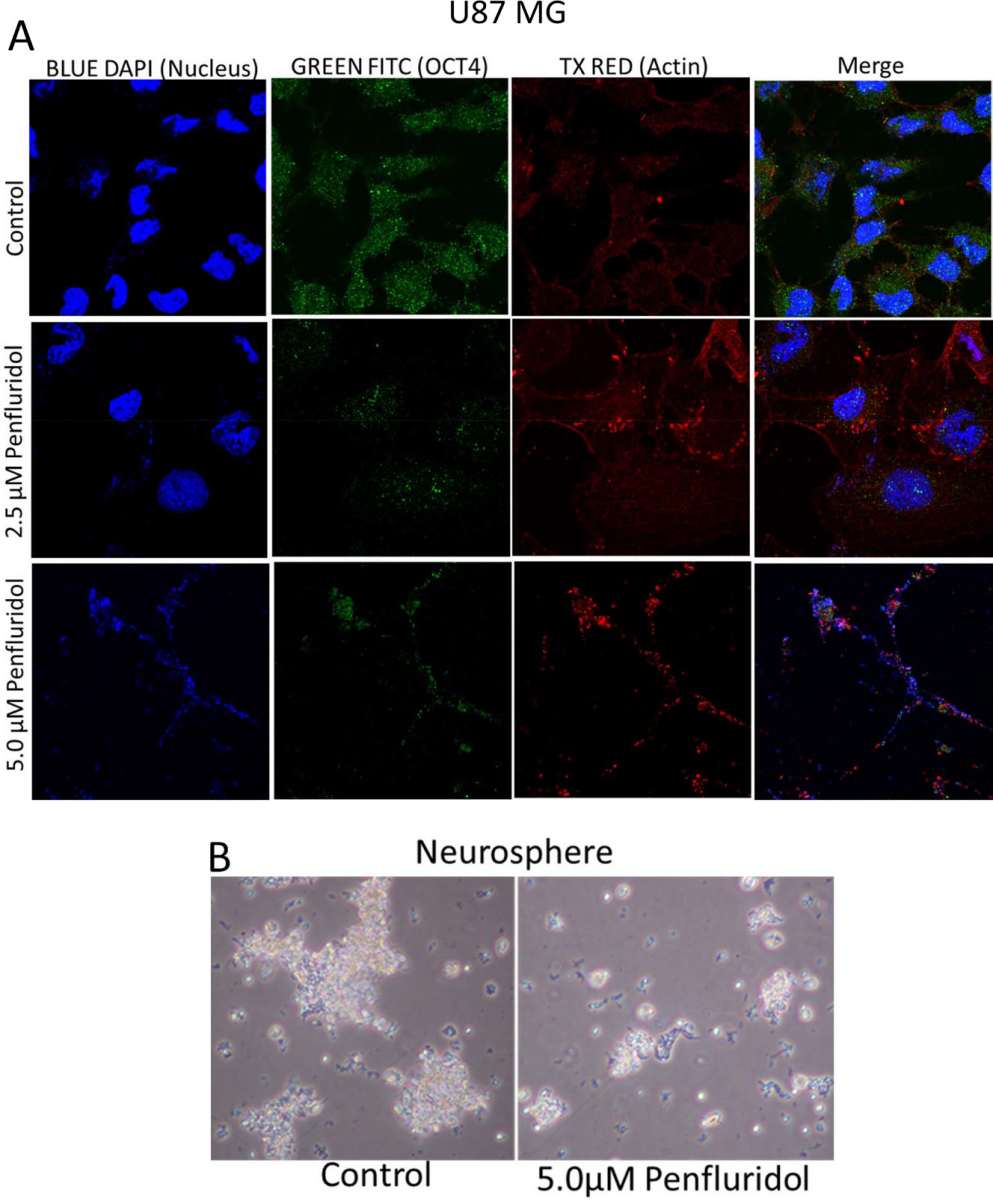
Penfluridol treatment suppresses OCT4, a stem cell marker in glioblastoma cells and growth of neurosphere cells (**A**) About 0.1 × 10^6^ U87MG cells were plated in 24 well plate on a coverslip. Cells were treated with 2.5 μM and 5.0 μM penfluridol for 48 h. Cells were processed, mounted on slides and images were taken using multi photon confocal microscope. Blue florescence represents DAPI, green represents OCT4, whereas red represents actin. (**B**) About 0.1 × 10^6^ neurosphere cells were plated in an uncoated 6 well plate. Cells were treated with 5.0 μM penfluridol for 48 h. After 48 h, images of the plate were taken under light microscope.

To elucidate the molecular mechanism of the growth suppressive effects of penfluridol, we performed western blot analysis using whole cell lysates from GBM28, U87MG, SJ-GBM2 and CHLA-200 cells treated with 0, 1.25, 2.5, 5 and 7.5 μM penfluridol for 48 h. Our results showed that penfluridol treatment significantly reduced the phosphorylation of Akt at Ser473 and the expression of GLI1 in a concentration-dependent manner in all the cell lines tested (Figure 3A–3C and Supplementary Figure 1). Protein expression of Akt was also reduced by treatment with 7.5 μM penfluridol in GBM28, SJ-GBM2 and CHLA-200 cells (Figure 3A–3B and Supplementary Figure 1). In addition, we observed a notable inhibition of the markers of cancer stem cells such as OCT4, Sox2 and Nanog. Furthermore, significant cleavage of caspase 3 and PARP was observed in a concentration-dependent manner in glioblastoma cells after 48 h of penfluridol treatment, indicating apoptosis (Figure 3A–3C and Supplementary Figure 1). Suppression of OCT4 by penfluridol treatment was also confirmed by immunofluorescence (Figure 4A). In addition, reduction in the growth of glioblastoma neurosphere cells was observed with 5 μM penfluridol treatment (Figure 4B).

### GLI1 silencing enhances the effects of penfluridol

**Figure 5:**
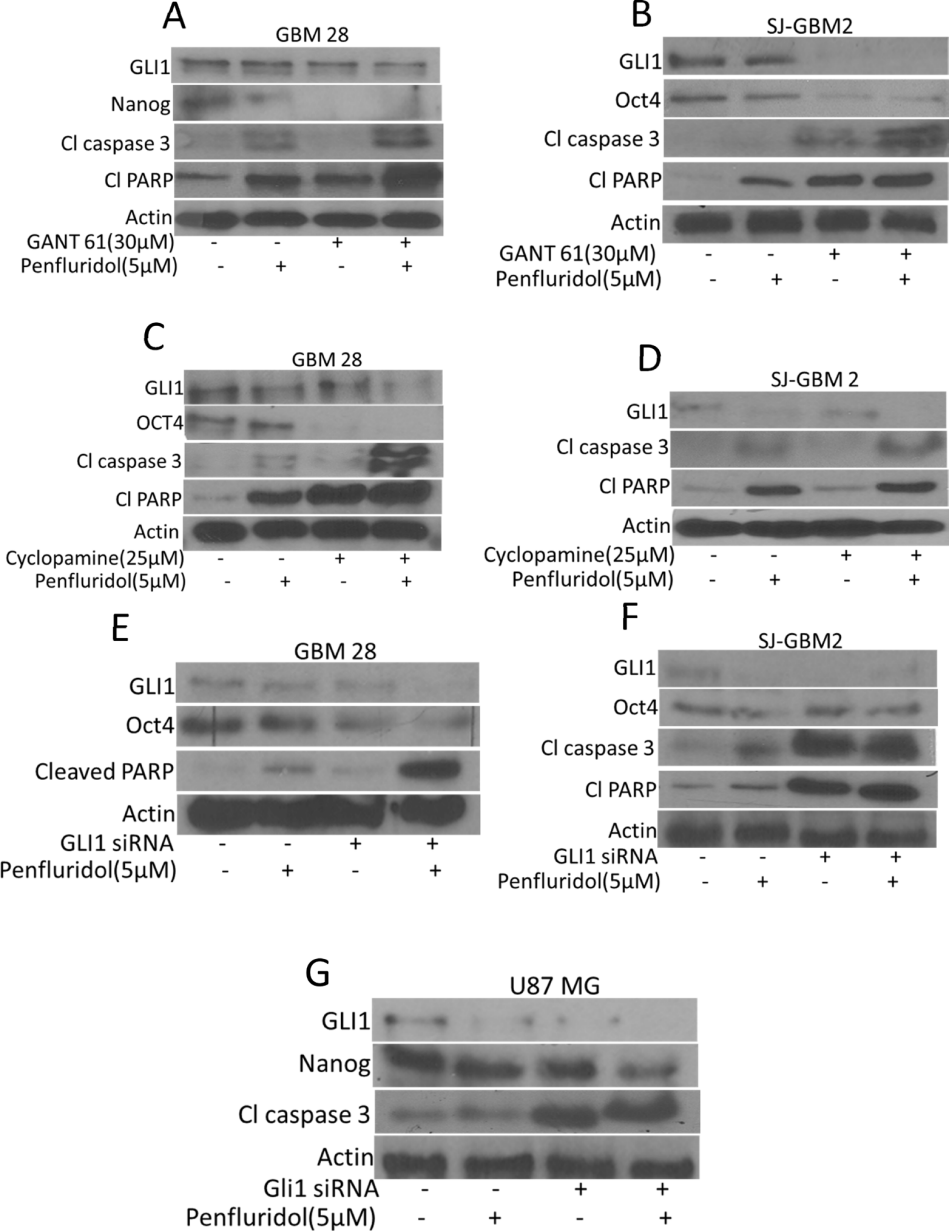
GLI1 mediated suppression of glioblastoma cell growth by penfluridol treatment GBM28 and SJ-GBM2 cells were treated with penfluridol (5 μM) for 48 h after (**A**–**B**) cells were pretreated with 30 μM GANT61 for 3 h (**C**–**D**) cells were pretreated with 25 μM Cyclopamine for 3 h (**E**–**G**) GBM28, SJ-GBM2 and U87MG cells were transfected with GLI1 siRNA. Actin was used as a loading control. Figures shown are the representatives blots of three independent experiments.

**Figure 6:**
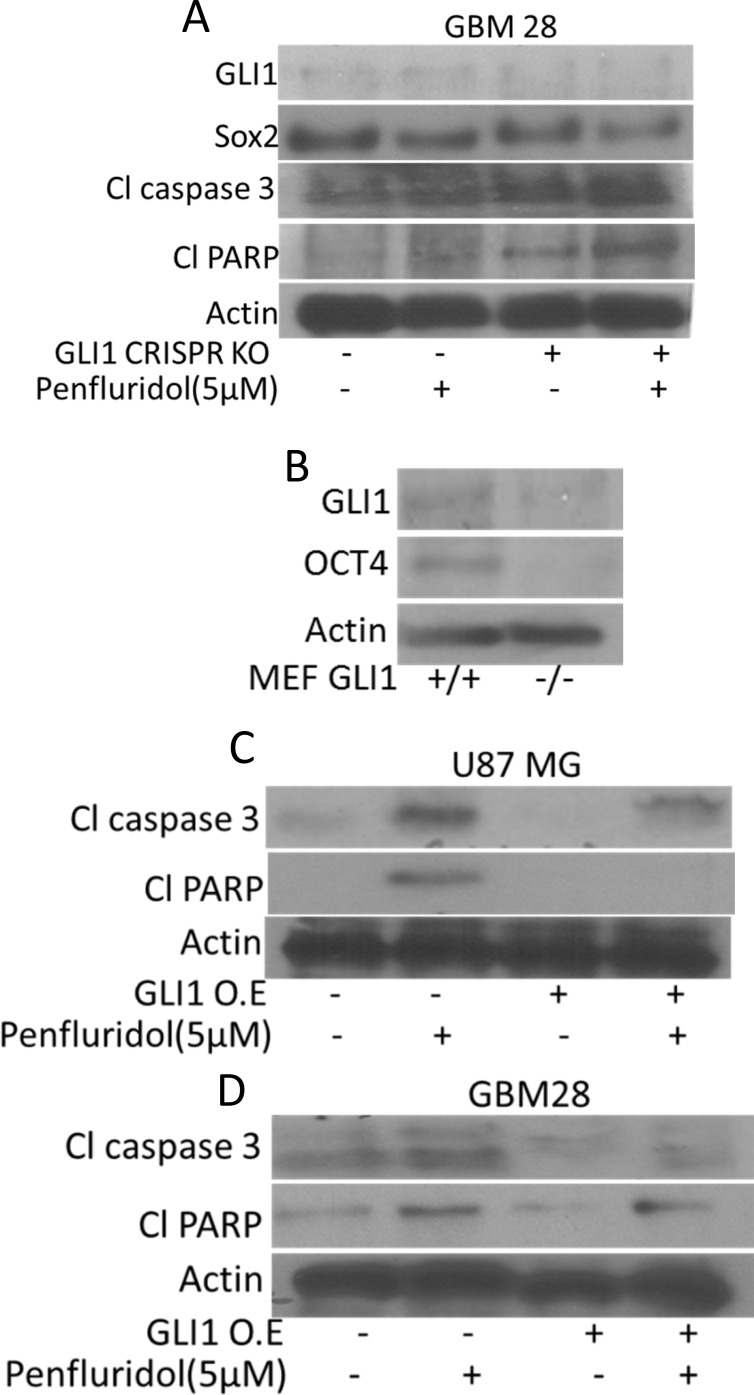
GLI1 mediated suppression of glioblastoma cell growth by penfluridol treatment (**A**) GBM 28 cells were transfected with GLI1 CRISPR/Cas9 KO (**B**) Levels of OCT4 were evaluated in GLI1 (+/+) as well as GLI 1(−/−) MEFs (Mouse embryonic fibroblasts). (**C**–**D**) U87MG and GBM 28 cells were transfected with GLI1 overexpressing plasmid. Levels of Cl caspase 3 and Cl PARP were evaluated by western blotting. Actin was used as a loading control. Figures shown are the representatives blots of three independent experiments.

To establish GLI1 as a target of penfluridol in glioblastoma, GLI1 was inhibited by pharmacological inhibitors or knocked down by GLI1 siRNA or CRISPR/Cas9. Effect of penfluridol in suppressing GLI1 was substantially enhanced in GBM28, SJ-GBM2 and U87MG cells in which, GLI1 was either inhibited by specific inhibitors or knocked down genetically (Figure 5A–5G) and (Figure 6A). Significant cleavage of caspase 3 as well as PARP was observed with penfluridol treatment in cells pretreated with GANT 61, cyclopamine or with GLI1 knock down using siRNA (Figure 5A–5G). Interestingly, enhanced inhibition of stem cell markers like OCT4 and Nanog, was also observed with penfluridol treatment in glioblastoma cells pretreated with GANT61, cyclopamine or GLI1 knock down using siRNA (Figure 5A–5G). Nonetheless, transfection of GBM 28 cells with GLI1 CRISPR/Cas9 resulted in enhanced cleavage of caspase 3 and PARP with penfluridol treatment (Figure 6A). These results indicated GLI1 mediated growth suppressive effect of penfluridol in glioblastoma cells.

### GLI1 regulates stem cell properties in glioblastoma cells

Inhibition of GLI1 by established inhibitors like GANT61 or cyclopamine or knocking down GLI1 using siRNA or GLI1 CRISPR/Cas9 significantly reduced the expression of OCT4 and Nanog indicating direct regulation of cancer stem cells by GLI1 in glioblastoma cells (Figure 5A–5G) and (Figure 6A). To further confirm these observations, we determined the expression of OCT4 in wild type and GLI1 KO mouse embryonic fibroblasts (MEF). Our results demonstrated significantly low expression of OCT4 in GLI1 (−/−) MEFs as compared to GLI1 (+/+) MEFs (Figure 6B). These results indicate that GLI1 regulates stem cells properties in glioblastoma cells.

### Overexpression of GLI1 abrogates the growth suppressive effects of penfluridol

To further establish GLI1 as a target of penfluridol, we overexpressed GLI1 using GLI1 overexpressing plasmid. Reduced cleavage of caspase 3 and PARP was observed with penfluridol treatment when GLI1 was overexpressed, indicating that penfluridol induces apoptosis and suppresses the growth of GBM cells through GLI1 inhibition, establishing GLI1 as a target of penfluridol (Figure 6C–6D).

### Akt regulates penfluridol mediated inhibition of GLI1

**Figure 7:**
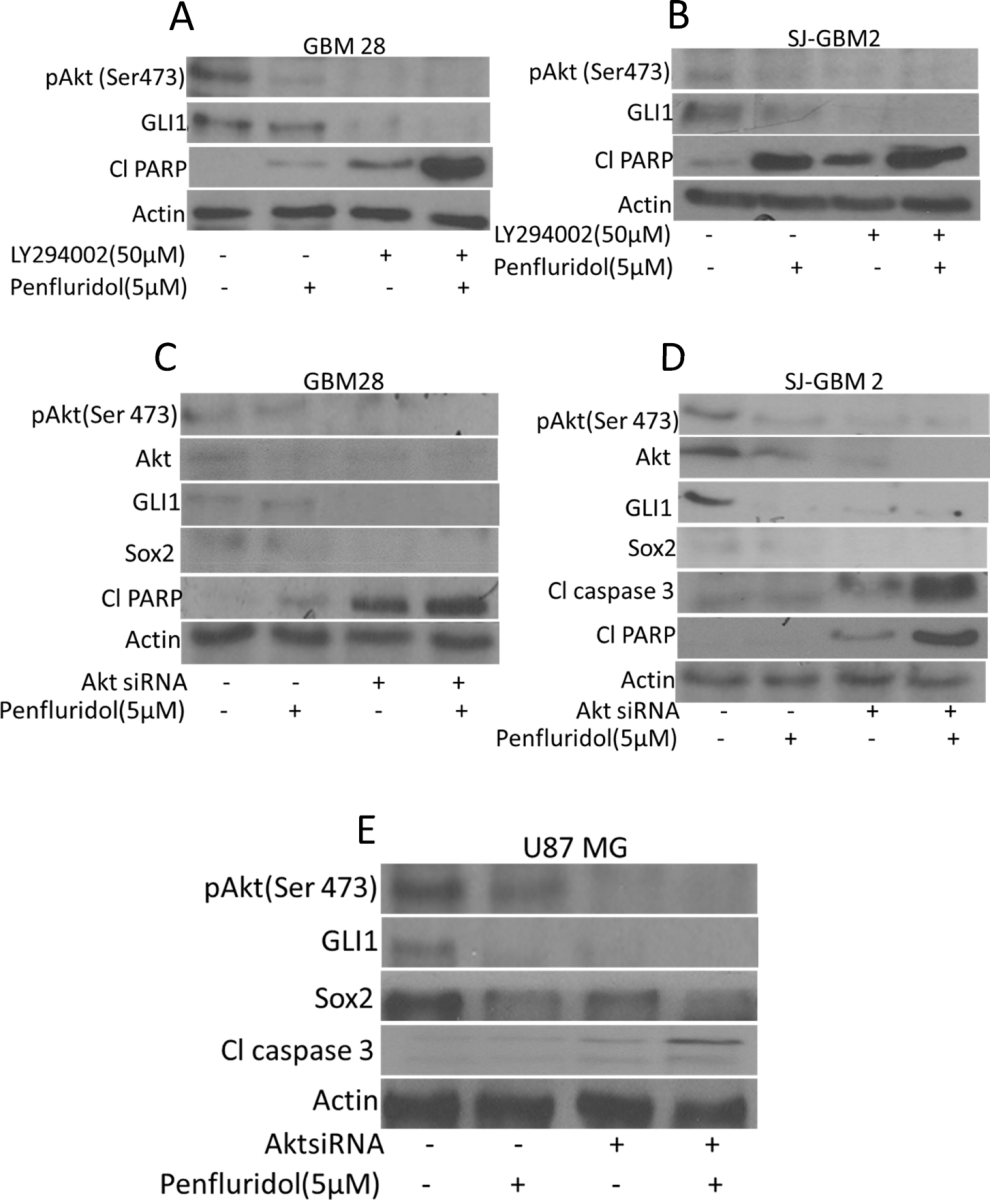
Akt mediated suppression of GLI1 by penfluridol treatment in glioblastoma cells GBM28, SJ-GBM2 and U87MG cells were treated with penfluridol (5 μM) for 48 h after (**A**–**B**) GBM28 and SJ-GBM2 cells were pretreated with 50 μM LY294002 (**C**–**E**) GBM28, U87MG and SJ-GBM2 cells were transfected with AktsiRNA. Levels of pAkt(Ser 473), GLI1, Sox2, Cl caspase3 as well as Cl PARP were evaluated by western blotting.

Akt (Protein Kinase B) is a serine/threonine protein kinase that plays a key role in multiple cellular processes including apoptosis and cell proliferation and is also required for maintenance of glioblastoma growth. Our results showed that penfluridol treatment inhibited the activation of Akt by suppressing its phosphorylation at Ser473 (Figure 3A–3C). To establish whether Akt regulate GLI1, glioblastoma cells were treated with PI3K/Akt inhibitors or Akt was knocked down using siRNA. Interestingly, inhibiting the phosphorylation of Akt using LY294002, a PI3K/Akt inhibitor or knocking down Akt using Akt siRNA, resulted in reduced expression of GLI1 (Figure 7A–7E). Enhanced cleavage of caspase 3 and PARP with penfluridol treatment was observed in SJ-GBM2, GBM28 and U87MG cells where Akt was inhibited (Figure 7A–7E). We also observed reduced expression of Sox2 with penfluridol treatment in the cells where Akt was silenced using AktsiRNA (Figure 7C–7E). To further delineate the mechanism of GLI1 inhibition through Akt by penfluridol treatment, effect of penfluridol on Akt interaction with GLI1 was evaluated by immunoprecipitation studies. GLI1 expression was clearly visible in GBM28 cells immunoprecipitated with pAkt (Ser473) indicating direct association or regulation of GLI1 by Akt. Nonetheless, this association of Akt with GLI1 was reduced significantly by penfluridol treatment (Data not shown).

### Penfluridol suppresses the growth of glioblastoma *in vivo*

**Figure 8:**
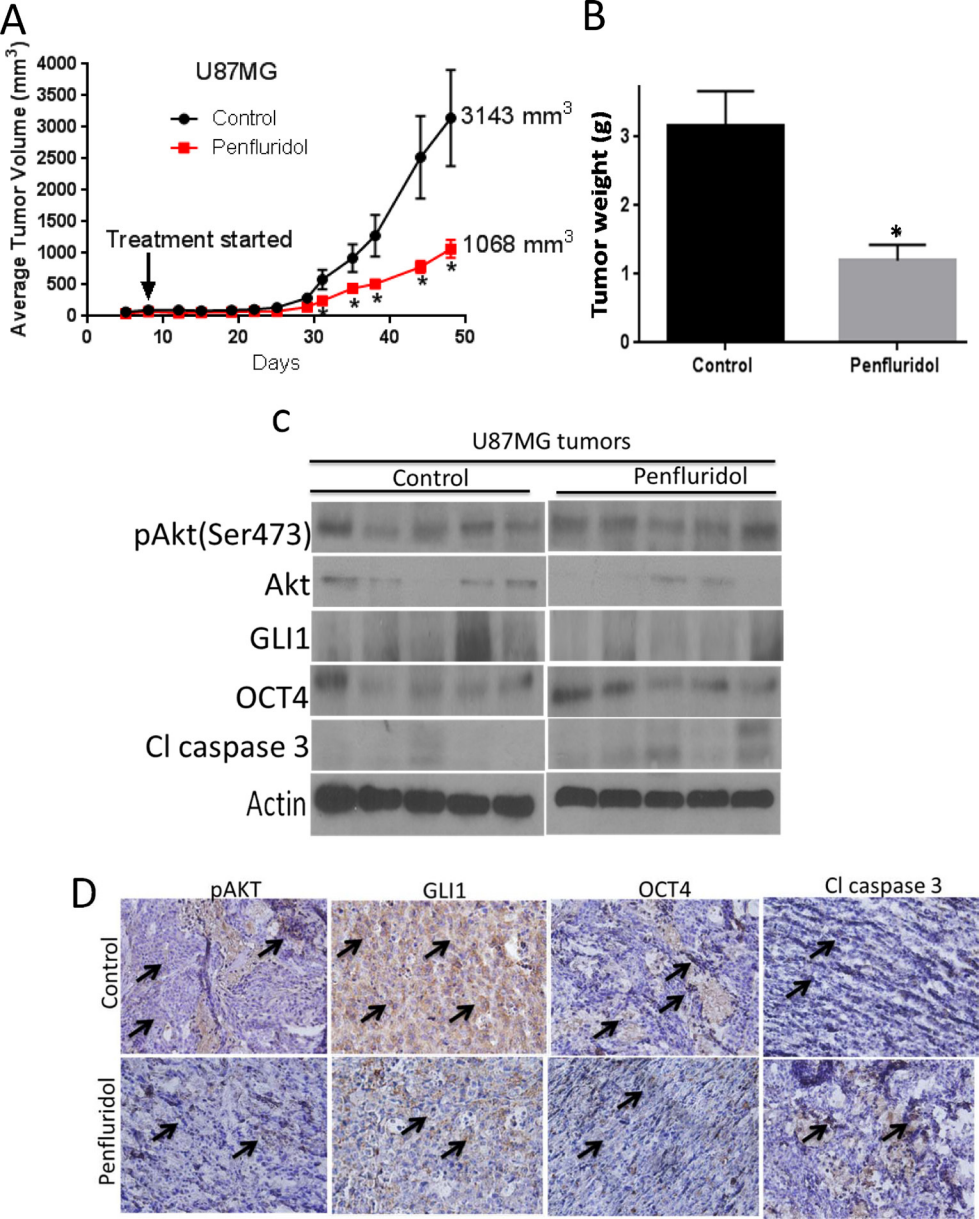
Penfluridol suppresses the growth of glioblastoma tumors through inhibition of Akt-GLI1 signaling in U87MG subcutaneous model (**A**) 1 × 10^6^ U87MG cells in 1:1 mixture of PBS and matrigel were implanted in right and left flanks of 4–6 week old athymic nude mice (*n* = 5). Treatment with 10 mg/kg penfluridol by oral gavage everyday started once tumor volume was 70-100 mm^3^ till day 48. Values were plotted as mean ± SEM. (**B**) Tumors were weighed once isolated from control and penfluridol-treated mice. Values were plotted as mean ± SEM. Tumors were removed after terminating the experiment, homogenized, lysed, and analyzed for pAkt(Ser473), GLI1, OCT4 and Cl caspase3 by western blotting. Actin was used as loading control. (**C**) Each lane of blot represents tumor from individual mouse. (**D**) Tumors were sectioned and immunostained for pAKT, GLI1, OCT4 and Cl caspase 3 as described in materials and methods.

To test the efficacy of penfluridol in inhibiting the growth of glioblastoma *in vivo*, U87MG adult glioblastoma cells were implanted subcutaneously in right and left flanks of athymic nude mice. Once each mouse attained about 70mm^3^ tumor, mice were randomly divided into two groups. Experimental group of mice were treated with 10 mg/kg penfluridol by oral gavage everyday whereas mice in control group received vehicle only. Due to excessive tumor burden in control group, mice were sacrificed at day 48. At the day of sacrifice, average tumor volume of 3,143mm^3^ was observed in control group as compared to 1,068mm^3^ in penfluridol treated group, indicating 65% suppression of glioblastoma tumor growth by penfluridol treatment (Figure 8A). After terminating the experiment, tumors were removed and weighed. Average weight of penfluridol treated tumors was about 68% less than the tumors from control group (Figure 8B). Tumors were analyzed by western blot analysis and immunohistochemistry. In agreement with our *in vitro* findings, we observed down regulation of GLI1 and increased cleavage of caspase 3 in the tumors from penfluridol treated mice as compared to tumors from control mice (Figure 8C). In Figure 8C, each band represents lysate from separate tumor coming from separate mouse. However, modest decrease in pAkt(Ser 473) and OCT4 level was observed in the tumors from penfluridol treated mice as evaluated by immunohistochemistry (Figure 8D). Our immunohistochemical results further demonstrated significantly reduced expression of GLI1 and increased cleavage of caspase 3 in tumors from penfluridol treated mice (Figure 8D).

In a separate experiment, SJ-GBM2, pediatric glioblastoma cells were implanted subcutaneously in athymic nude mice. Mice were treated with 10 mg/kg penfluridol by oral gavage everyday once each mouse had palpable tumor. Penfluridol treatment suppressed the growth of pediatric glioblastoma tumors by 35% as compared to control group (Supplementary Figure 2A). Surprisingly, effect of penfluridol in inhibiting the growth of pediatric glioblastoma was less as compared to what we observed with adult glioblastoma. This could be attributed to the fact that H3F3A mutations are common in pediatric glioblastoma, whereas IDH1 mutations are common in adult glioblastoma tumors. Tumors collected after sacrificing the mice were subjected to western blot analysis, and immunohistochemistry. In agreement with our *in vitro* observations in SJ-GBM2 cells, we observed reduced pAkt(Ser473) and GLI1, and increased cleavage of caspase 3 with penfluridol treatment (Supplementary Figure 2B). These observations were further confirmed by immunohistochemical analysis in control and penfluridol treated tumors where similar observations were made (Supplementary Figure 2C). These results indicated that penfluridol suppresses glioblastoma tumor growth by Akt-mediated inhibition of GLI1.

**Figure 9:**
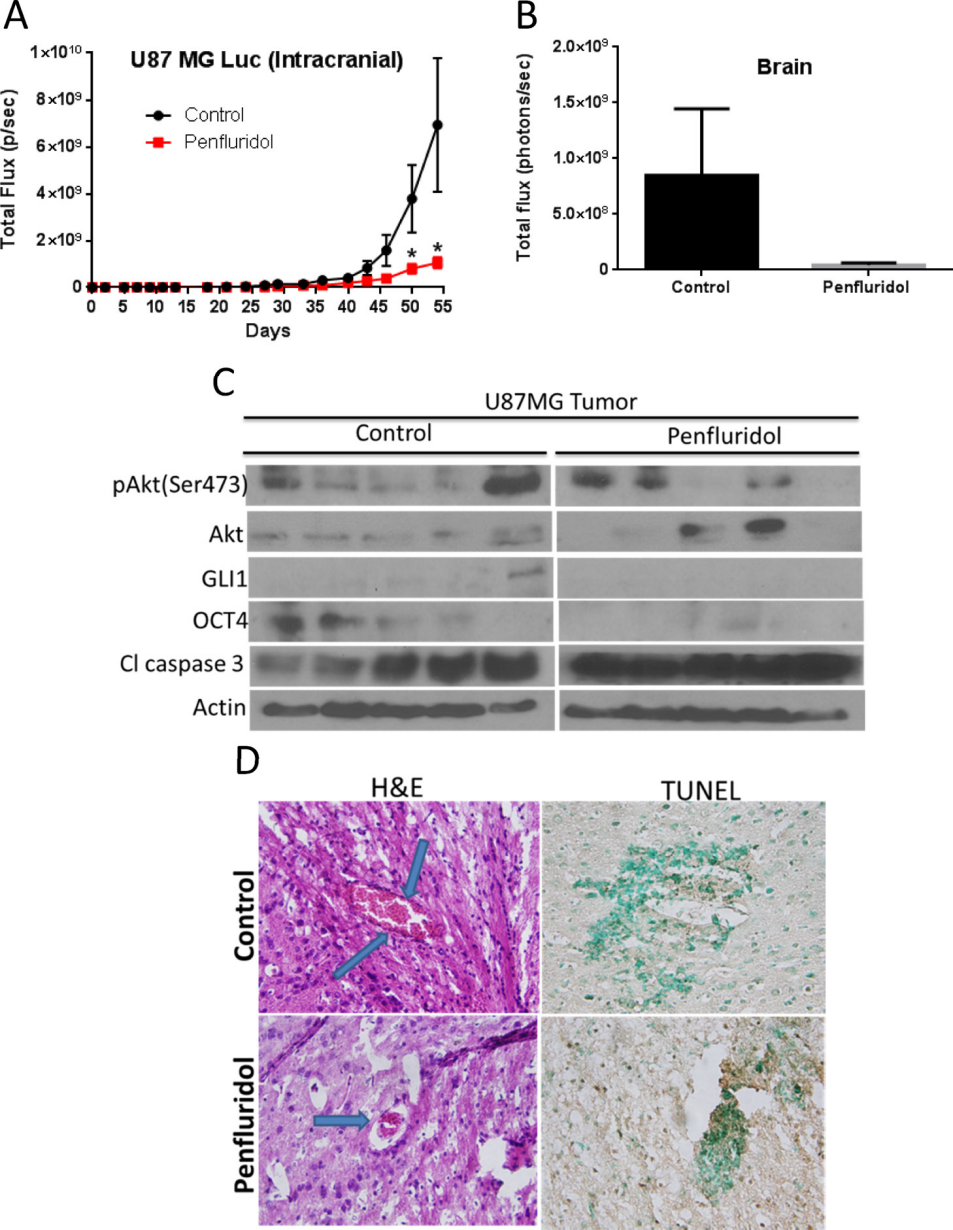
Penfluridol suppresses the growth of intracranially implanted U87MG brain tumors About 0.15 × 10^6^ U87MG-luc glioblastoma cells were injected in the brain of 4 to 6-week old female athymic nude mice using stereotaxic apparatus (*n* = 11). 10 mg/kg penfluridol by oral gavage was given to each mouse everyday till day 54 starting day 14 after tumor cells implantation after each mouse showed significant luminescence in the brain. (**A**) Luminescence in brain total flux (photons/sec) of all the control and treated group mice was plotted till day 54. Values were plotted as mean ± SEM. (**B**) At day 54, experiment was terminated and brain from control and penfluridol-treated mice were removed, imaged, and luminescence of control and penfluridol-treated group was plotted. Values were plotted as means ± SEM. Few brains with tumors were homogenized, lysed and analyzed for pAkt(Ser473), GLI1, OCT4 and Cl caspase3 by western blotting. Actin was used as loading control. (**C**) Each lane of blot represents tumor from individual mouse. Brains were sectioned, processed, and immunostained for (**D**) H&E and TUNEL.

As a proof-of-principle and to further confirm the efficacy of penfluridol in inhibiting the growth of glioblastoma tumors in brain, U87MG-luc cells were injected intracranially into the brain of mice using steriotaxic apparatus. Mice were treated with 10 mg/kg penfluridol by oral gavage every day after 14 days of tumor cell implantation in the brain. Weight of mice was taken once a week. No significant change in the average weight of mice in control and penfluridol treated group was observed (Supplementary Figure 3). Our results showed that penfluridol treatment suppressed the growth of intracranially implanted glioblastoma tumors in brain by 72% (Figure 9A). We also analyzed luminescence in the isolated brains from both the groups after euthanizing the mice. Average brain luminescence from penfluridol treated group was 94% less as compared to control group (Figure 9B). The isolated brains with tumors were lysed for western blot analysis. In agreement with *in vitro* observations, penfluridol treatment reduced pAkt(Ser473), GLI1 and OCT4, and enhanced cleavage of caspase 3 in brain tumors (Figure 9C). As evaluated by TUNEL, significant apoptosis was observed in the brain with glioblastoma tumors by penfluridol treatment (Figure 9D). H & E staining of brain tumors clearly showed smaller tumor size in the treatment group as compared to control (Figure 9D).

To study the long term effect of penfluridol administration, locomotor activity was evaluated using Versamax chamber in mice implanted with tumors in the brain and treated with 10 mg/kg penfluridol everyday by oral gavage for 54 days. No significant difference in the locomotor activities of mice such as total distance, horizontal activity, ambulatory activity, rest time, locomotor clockwise revolution and locomotor counter clockwise revolution were observed between control and penfluridol treated group (Supplementary Figure 4A–4F). These results indicated that chronic administration of penfluridol for treating glioblastoma tumors may not induce any significant behavioral change in mice activities. However, further in-depth detailed studies are required.

## DISCUSSION

Our current study demonstrated potential anti-cancer effects of penfluridol *in vitro* and *in vivo* against glioblastoma, which is known to be an incurable type of brain tumor. Penfluridol is an FDA-approved anti-psychotic drug specifically prescribed for schizophrenia. Thioridazine, another antipychotic drug was recently shown to inhibit the growth of glioblastoma by inducing autophagy [6]. However, thioridazine was associated with significant cardiac toxicity, thus preventing its use in the clinic [7].

In the current study, we evaluated the anti-cancer effects of penfluridol in a panel of ten different glioblastoma cell lines including patient-derived adult and pediatric cell lines. We observed that penfluridol treatment significantly reduced the viability of all the glioblastoma cells in a concentration and time-dependent manner with IC50 ranging between 3–5 μM after 48 h of treatment. Significant apoptosis was observed in all the cell lines treated with penfluridol. Several recent studies demonstrated the role of sonic hedgehog signaling, specifically GLI1 in glioblastoma progression and the role of GLI1 in imparting stem cells characteristics [2]. Interestingly, GLI1 was found to be up-regulated in glioblastoma and responsible for chemo-resistance [9, 10]. We observed that penfluridol treatment reduced the expression of GLI1 in all the glioblastoma cells in a concentration-dependent manner. Recently, it was shown that Akt signaling is required for maintenance of glioblastoma tumors [11] and non-canonical activation of GLI1 was observed by PI3K/Akt signaling in renal cell carcinoma [4]. However, it was not known whether GLI1 activation was mediated by Akt in glioblastoma. Our current studies established regulation of GLI1 by Akt in glioblastoma cells. Our results also demonstrated that penfluridol treatment suppressed the activation (phosphorylation) of Akt in a concentration-dependent manner in all the glioblastoma cell lines tested.

It has been reported that glioblastoma tumors develop resistance to temozolomide therapy due to overexpression of GLI1, and that GLI1 imparts stem cell like properties to cancer cells rendering them ineffective to chemotherapy [1]. Furthermore, initiation and maintenance of cancerous phenotype requires intricate and synergistic interaction with several oncogenic signaling [12]. In our current study, we demonstrated that penfluridol treatment inhibited the growth of glioblastoma cells by Akt-mediated inhibition of GLI1. OCT4, Sox2 and Nanog are basic transcriptional factors that are expressed in cancer stem cells as well as in embryonic stem cells [13]. Upregulation of OCT4, Sox2 and Nanog has been reported in many cancers such as prostate and breast cancer [14] [15]. Also, these transcription factors are overexpressed in poorly differentiated tumors than in tumors that are well differentiated [14]. Our results showed down regulation of cancer stem cell markers such as OCT4, Nanog and Sox2 by penfluridol treatment. Inhibiting GLI1 with commercially available inhibitors like GANT61 and cyclopamine or knocking down using GLI1siRNA and GLI1 specific CRISPR/Cas9, significantly enhanced the growth suppressive effects of penfluridol. Of note, our studies indicated that knocking down GLI1 reduced the expression of OCT4 and Nanog showing direct regulation of cancer stem cells by GLI1 in glioblastoma cells. On the other hand, overexpression of GLI1 using GLI1 specific plasmid, reduced the growth inhibitory effects of penfluridol, indicating the specificity of penfluridol for GLI1 in glioblastoma cells. Interestingly, inhibiting Akt using LY294002 or knocking down Akt using Akt specific siRNA resulted in not only down regulation of GLI1 but also enhanced the growth suppressive effects of penfluridol in our model, indicating the regulation of GLI1 by Akt.

Recently, we published that penfluridol significantly suppresses the metastatic tumor growth of breast cancer in brain by inhibiting integrinα6β4 signaling, supporting our rationale for current studies [8]. Repression of integrins with penfluridol treatment in breast cancer was also shown through reactive oxygen species and Sp transcription factors in a very recent study [16]. It has been reported by several published studies that GLI1 regulates integrins in different cancer models [17, 18]. Anti-cancer activity of penfluridol was also observed by Chien et al. in pancreatic cancer cells with IC_50_ ranging from 9–37 μM [19] while our current studies showed much lower IC_50_ in glioblastoma cells ranging 3–5 μM only. Interestingly, Wu et al. demonstrated anti-tumor activity of penfluridol through dysregulation of cholesterol whereas our previous studies showed autophagy mediated cell death by penfluridol in pancreatic cancer [20, 21].

We demonstrated previously that 10 mg/kg/day dose of penfluridol by oral gavage significantly inhibited the growth of metastatic breast tumors in the brain and orthotopically implanted tumors in the pancreas [8, 20]. In the current study, similar dose of penfluridol by oral gavage significantly suppressed the growth of glioblastoma tumors in three different glioblastoma tumor models. Our *in vivo* results showed 65% growth suppression of U87MG xenografts in athymic nude mice. As a proof of principle, in intracranial tumor model, we observed 72% growth inhibition of tumors in the brain by penfluridol treatment. Although, penfluridol, an antipsychotic drug has blood brain barrier penetrability, interestingly we observed almost similar efficacy of penfluridol in subcutaneous as well as intracranial model of glioblastoma. Future investigations with regard to the availability of penfluridol in peripheral tissues versus brain are thus required. Interestingly, similar dose of penfluridol was less effective in inhibiting the growth of SJ-GBM2, pediatric glioblastoma tumors in xenograft model. Low efficacy of penfluridol against pediatric tumor may in part be attributed to the fact that one third of pediatric glioblastoma is characterized by H3F3A mutations, whereas IDH1-mutated glioblastoma is common in adult patients [22]. H3F3A mutation in pediatric glioblastoma drives the upregulation of oncogenes [23]. Our results on the efficacy of penfluridol in adult GBM cells with IDH1 mutations are supported by findings from other laboratories, which shows that IDH1 mutation is associated with prolonged overall survival and better efficacy of temozolomide [24]. On the other hand, H3F3A mutation is associated with poor prognosis in children [25] and long term survivors are associated with wild type H3F3A [25]. Critical molecular differences between adult and pediatric glioblastoma does not guarantee similar efficacy of a given drug in both the cases [26]. Nonetheless, further in-depth studies are required to establish the reason behind differential efficacy of penfluridol in adult and pediatric glioblastoma. Consistent with our *in vitro* observations, we observed reduced phosphorylation of Akt at Ser473 and expression of GLI1 and OCT4, as well as cleavage of caspase 3 and PARP, in the tumors from penfluridol treated mice as compared to control mice, as evaluated by western blotting and immunohistochemistry. Penfluridol induced apoptosis was confirmed by TUNEL staining. No general toxicity or behavioral side effect was observed in the brain tumor bearing mice treated with 10 mg/kg/day penfluridol as compared to control mice.

Overall, our studies established the anti-tumor efficacy of penfluridol against glioblastoma via Akt-mediated suppression of GLI1. To the best of our knowledge, this is the first report showing suppression of glioblastoma tumor growth by penfluridol treatment, setting the foundation for developing penfluridol as a treatment option for glioblastoma patients.

## MATERIALS AND METHODS

### Ethics statement

All the animal experiments were conducted in accordance with the ethical standards and according to approved protocol by Institutional Animal Care and Use Committee (IACUC), Texas Tech University Health Sciences Center.

### Cell culture

Patients-derived glioblastoma cells GBM43, GBM10, GBM44, GBM28, GBM14 as well as U251MG used in this study were kindly provided by Dr. Jann N. Sarkaria, Mayo Clinic, Rochester, Minnesota. GBM28 and GBM43 have mutated p53 whereas p16 is deleted in GBM10, GBM14, GBM43 and GBM44 [27]. Patients-derived glioblastoma cells were maintained in DMEM supplemented with 10% FBS and 1% PSN whereas U251MG cells were maintained in EMEM media supplemented with 10% FBS and 1% PSN. U87MG and T98G cells were purchased from ATCC, Manassas, VA, and maintained in EMEM supplemented with 10%FBS and 1% PSN. We started working with U87MG cell line 2–3 years ago when there was no question regarding its origin. Unfortunately, recently, origin of U87MG was found to be different than from the reported GBM tumor. However, it is still established that the origin of U87MG is from tumors present in the brain. Pediatric patients-derived glioblastoma cells, SJ-GBM2 and CHLA-200 used in this study were kind gift from Dr. Patrick Reynold, Children Oncology Group (COG), Texas Tech University Health Sciences Center. SJ-GBM2 cells were derived from a five year old female patient who relapsed after chemotherapy, whereas CHLA-200 was derived from a twelve year old male patient after post-mortem who relapsed after chemotherapy. Cells were maintained in IMDM media supplemented with 20% Fetal Bovine Serum, 4 mM L-Glutamine, 1X ITS (5 μg/mL insulin, 5 μg/mL transferrin, 5 ng/mL selenous acid). Mouse embryonic fibroblast MEF GLI1+/+ and MEF GLI1−/− were provided by Dr. Jann N. Sarkaria, Mayo Clinics, Rochester. MEFs were maintained in DMEM supplemented with 10%FBS and 1%PSN as described by us previously [28]. Neurosphere forming cells, which form neurosphere were kind gift from Dr. Jann N. Sarkaria. For neurosphere formation, cells were plated on laminin coated plate and maintained in serum free media consisting of Knockout DMEM/F12 media supplemented with 2% serum free media supplement, 2% L-glutamine, 1% penicillin/streptomycin, 20 ng/ml EFG and 20 ng/ml FGF, as described previously [29].

### Cytotoxicity studies

Cells were plated at a density of about 3000–5000 cells/well in 96 well plates and incubated overnight. Next day, cells were treated with different concentrations of penfluridol (Sigma-Aldrich, St. Louis, MO). After desired duration of treatment with penfluridol (24, 48 and 72 h), cells were fixed using ice cold 10% trichloroacetic acid followed by washing with water and staining with Sulforhodamine B (SRB) dye. Plates were washed with 1% solution of acetic acid and the optical density was measured in 10 mM Tris-base solution, using plate reader (BioTek Instruments, VT) as described by us previously [30].

### AnnexinV/FITC apoptosis assay

AnnexinV/FITC apoptosis assay was performed using a commercially available kit and according to manufacturer's instruction (BD Biosciences, San Jose, CA, USA). Approximately 0.2–0.3 × 10^6^ GBM cells were plated in each well of 6 well plate and left overnight in the incubator. Penfluridol was added at required concentrations. After 48 h of treatment, cells were trypsinized and suspended in PBS. Cells were washed in PBS and suspended in 150 μl of binding buffer. 4 μl Annexin-V FITC and 4 μl propidium iodide was added followed by incubation for 20 minutes in dark. Volume was adjusted to 300 μL by adding binding buffer. Samples were analyzed by flow cytometer after mixing the cells gently (Accuri C6, Ann Arbor, MI, USA) and as described by us previously [20].

### Western blot analysis

The whole cell lysates were prepared using 4% (w/v) CHAPS in urea-tris buffer. 20–60 μg of protein obtained after lysing the cells were subjected to SDS-PAGE and the resolved proteins were transferred to PVDF membrane. The membranes were probed for primary antibodies against pAkt(Ser473), GLI1, OCT4, Nanog, Sox2, Cl PARP, Cl caspase 3 and Actin. All primary antibodies were purchased from Cell Signaling Technologies (Danvers, MA) except pAkt(Ser473) (Santa Cruz Biotechnology, Inc., Dallas, TX) and Actin (Sigma Aldrich, St. Louis, MO). Dilution factor was 1:1000 for all the antibodies from Cell Signaling and 1:2000 for Actin antibody. The membranes were developed as described by us previously [8, 31, 32].

### Sonic hedgehog inhibitors treatment

GBM28, SJ-GBM2 and U87MG glioblastoma cells were plated at a density of 0.2–0.3 × 10^6^ cells per well in a six-well plate. After overnight incubation, cells were pretreated for 3h with 25 μM cyclopamine or 30 μM GANT 61. Cyclopamine and GANT 61 were purchased from Cayman Chemical, Ann Arbor, MI. After pretreatment with inhibitors, cells were treated with 5 μM penfluridol for 48 h and processed for western blotting as explained above.

### Silencing GLI1

GBM28, SJ-GBM2 and U87MG cells were transfected with GLI1 siRNA (Santa Cruz Biotechnology, Inc, Dallas, TX) using siPORT (Ambion Inc, Austin, TX) transfection reagent as per manufacturer's protocol. Cells were transfected with 100 nM GLI1 siRNA and after 12 h post transfection, cells were treated for additional 48 h with 5 μM penfluridol. The cells were collected after treatment and processed for western blot analysis as described by us before [20].

### GLI1 knockout via CRISPR/Cas9

GBM 28 cells were plated at a density of 02–0.3 × 10^6^ cells per well in a six well plate. After overnight incubation and with 50–70% confluency, cells were transfected with gRNA, Origene Technologies (Rockville, MD) using TurboFectin, and as per manufacturer's instruction. GLI1 CRIPSR/Cas9 was purchased from Origene Technologies (Rockville, MD). 96 h post transfection, cells were treated with 5 μM penfluridol for 48 h after which cells were collected and processed for western blot analysis as described by us before.

### Akt inhibitor treatment

GBM28 and SJ-GBM2 cells were plated in a six well plate at a density of 0.2–0.3 × 10^6^ cells per well. Cells were left for overnight incubation. After incubation, cells were pretreated for 3 h with LY294002, known PI3K/Akt inhibitors. LY294002 was purchased from Cayman Chemicals (Ann Arbor, MI). After pretreatment with inhibitors, cells were treated with 5 μM penfluridol for 48 h and processed for western blotting as explained above

### Silencing of Akt

GBM 28, SJ-GBM2 and U87MG cells were transfected with Akt siRNA (Cell Signaling Technologies, Danvers, MA). Transfection was performed using siPORT (Ambion Inc, Austin, TX) transfection reagent and as per manufacturers protocol. Cells were transfected with 100 nM Akt siRNA and after 12 h post transfection, cells were treated for additional 48 h with 5 μM penfluridol. The cells were collected after treatment and processed for western blot analysis as described by us before [33].

### Immunoprecipitation

Immunoprecipitation was performed as described by us previously [34, 35]. Briefly, 0.7 × 10^6^ GBM 28 cells were plated in 100mm tissue culture dishes and treated with 5 μM penfluridol. After 48 h of penfluridol treatment, whole cell lysates were prepared using RIPA buffer and immunoprecipitated with pAkt(Ser473) antibody (Santa Cruz Biotechnology Inc, Dallas, TX). Immune complexes were resolved on SDS-PAGE and immunoblotted for GLI1 (Cell Signaling Technology, Denvers, MA).

### Immunofluorescence analysis

Immunofluorescence analysis was performed as described by us earlier [31, 34]. U87MG cells were plated in a 24-well plate on a coverslip at a density of 0.1 × 10^6^cells/well and allowed to attach overnight followed by treatment with 2.5 μM and 5 μM penfluridol for additional 48 h. The cells were fixed with formalin and Triton-X100 solutions to permeabilized the cells. After blocking with 6% goat serum in 1%BSA, cells were incubated overnight with antibody against OCT4 (1:400). Cells were washed with PBS and again incubated overnight with Actin antibody (1:2000). Next day cells were incubated with AlexaFluor 488 secondary antibody (Invitrogen, Carlsbad, CA) as well as AlexaFlour 594 secondary antibody (Invitrogen, Carlsbad, CA) separately, after washing three times with PBS in between and after incubation. Coverslips were mounted on slides (mounting media with DAPI) and images were taken using multi photon confocal microscope (Nikon).

### Neurosphere assay

Neurosophere of GBM12TMZ-3080RZ cells were maintained in knockout DMEM/F-12 media supplemented with SFM supplement, L-glutamine, penicillin-streptomycin, FGF and EGF. Neurosphere forming cells were plated on laminin coated culture plate. Neurospheres were collected from culture plate along with media. Neurosphere pellet was formed by centrifugation, re-suspended in Trypsin and placed in the incubator for three minutes. Pellet was pipetted to further break the neurospheres. Cells were counted and about 0.1 × 10^6^ cells per well were plated in an uncoated 6 well plate. After incubation, cells from neurosphere were treated with 5 μM penfluridol for another 48 h. Images were taken using light microscope (Leica).

### U87MG xenograft implantation

Athymic nude mice (4–6 week old) were purchased from Harlan Laboratory (Livermore, CA). The use and treatment of athymic nude mice was approved from Institutional Animal Care and Use Committee (IACUC), Texas Tech University Health Sciences Center, Amarillo, Texas. All the experiments were performed under the strict compliance and regulations. 1 × 10^6^ U87 MG cells in 1:1 mixture of PBS and matrigel were implanted in right and left flanks of mice. Once tumor volume reached around 70–100 mm^3^, mice were randomly divided into two groups with five mice in each group. Group I served as control and received the vehicle only. Group II received 10 mg/kg penfluridol by oral gavage every day. Penfluridol stock was made in DMSO, which was further diluted in water/PEG300/ethanol/2% acetic acid in 8:3:0.13:1 v/v [8]. Tumor volume was measured twice a week till day 48 by using vernier caliper as described by us before [36]. At day 48, mice were humanely sacrificed and tumors were removed. Weights of all the tumors were taken. A part of tumor was snap frozen for western blotting and other part was fixed in formalin for immunohistochemistry.

### Patient-derived SJ-GBM2 xenograft tumors

Female athymic nude mice (4–6 week old) were purchased from Harlan Laboratory (Livermore, CA). All the experiments were performed under the strict compliance and regulations as mentioned before. 1 × 10^6^ SJ-GBM2 cells in 1:1 mixture of PBS and matrigel were implanted in flanks of mice. Once tumor volume was around 50–70 mm^3^, mice were randomly segregated into two groups with five mice in each group. Test group of mice received 10 mg/kg penfluridol by oral gavage every day till day 39, whereas control mice received vehicle alone. Experiment was terminated at day 39 by euthanizing mice with CO_2_ overdose. The tumors were removed from each mouse, weight was taken and a part of tumor was snap frozen in liquid-nitrogen for western blot analysis. Another part of tumor was fixed in formalin for immunohistochemical analysis.

### Intracranial tumor model of U87MG tumors

Female athymic nude mice (4–6 weeks old) from Harlan Laboratories (Livermore, CA) were used for intracranial injection and the experiments were conducted in strict compliance with the regulations of Institutional Animal Care and Use Committee (IACUC), Texas Tech University Health Sciences Center. U87MG-luc cells were harvested, washed twice with sterile PBS and re-suspended in PBS at a density of 30 × 10^6^ cells per ml. A suspension of 5 μl containing 0.15 × 10^6^ cells was injected by intracranial route using stereotaxic apparatus. Following this, mice were randomly divided into two groups with eleven mice in each group. Penfluridol at the dose of 10 mg/kg was administered to mice by oral gavage after 14 days of tumor cells implantation in the brain and every day after that. The tumor growth was monitored by non-invasive *in vivo* live animal imaging (IVIS Caliper) as described before [37]. Experiment was terminated at day 54 by humanely euthanizing the mice with CO_2_ overdose and mice brain were carefully dissected out, imaged for luminescence. Few brains from control and penfluridol treated group mice were snap frozen for western blot analysis whereas other brains were fixed in formalin for immunohistochemical and TUNEL analysis.

### Immunostaining of tissue sections

The immunohistochemistry (IHC) was performed as previously described by us [36, 38]. Briefly, formalin fixed tumor tissues were dehydrated and embedded in paraffin. Tissues were sectioned into 5 μM thick sections using microtome (Leica Microsystems Inc., Buffalo Grove, IL). The sections were deparaffinized and rehydrated by performing three washes of xylene for 10 minutes each, two washes of 100% ethanol for 5 minutes each followed by two washes of 95% ethanol for 6 minutes and one wash each of 70% and 50% ethanol for 3 minutes. At last the sections were given two 5 minute washes in distilled water (dH2O). Unmasking of antigen was done by boiling the sections in 10mM sodium citrate buffer (pH 6.0). Sections were washed and incubated in 3% hydrogen peroxide solution. The sections were blocked with 6% goat serum in 1% BSA for half hours and incubated with primary antibodies for cleaved caspase 3 (1:300), pAkt(Ser473) (1:300), GLI1 (1:300) and OCT4 (1:300) overnight at 4°C. Next day the slides were stained using Ultravision ONE HRP polymer kit as per the manufacturer's instructions (Thermofisher scientific, Fremont, CA). Counterstaining of sections was performed with Mayer's hematoxylin and dehydrated. The slides were mounted using Permount (Fisher scientific, Fair Lawn, NJ) and imaged using Olympus microscope (Olympus America Inc, Center Valley, PA) at 20× magnification. Antibody for cleaved caspase 3 and OCT4 were purchased from Cell Signaling whereas antibody for GLI1 and pAkt(Ser473) used for immunohistochemistry were purchased from Santa Cruz Biotechnology, Inc., Dallas, TX. TUNEL assay was carried out according to manufacturer's protocol (TUNEL assay kit was purchased from Calbiochem, San Diego, CA, USA)

### Mice behavioral analysis

In intracranial experiment, mice from control and treated group were analyzed for any behavioral side effect due to penfluridol. Mice were observed for general signs of toxicity. After 54 days of treatment, behavioral activity of mice was assessed using Versamax (AccuScan Instruments Inc., Columbus, OH, USA) and as described by us before [8]. Versamax is a ventilated chamber equipped with infrared sensors along the side wall to monitor mice activity.

### Statistical analysis

Prism 6.0 software was used for all the statistical analysis (GraphPad software Inc., San Diego, CA). Results are represented as means ± standard deviation (SD) or standard error of means (SEM). Statistical significance was analyzed using Student's *t*-test and outcomes were considered statistically significant at *p* < 0.05.

## SUPPLEMENTARY MATERIALS FIGURES AND TABLES


