# Supplementary material for: Penfluridol suppresses glioblastoma tumor growth by Akt-mediated inhibition of GLI1
Source: Oncotarget. 2017 Mar 23;8(20):32960–76. doi: 10.18632/oncotarget.16515 (PMC5464842; doi:10.18632/oncotarget.16515)
Supplement: Supplementary file 1 [file oncotarget-08-32960-s001.pdf]

Penfluridol suppresses glioblastoma tumor growth by Akt-mediated inhibition of GLI1

Supplementary Materials

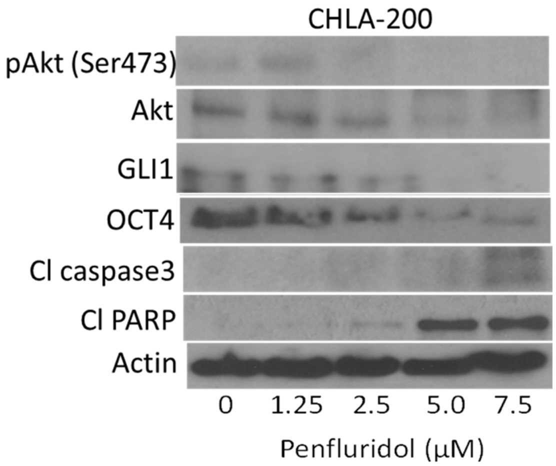

Supplementary Figure 1: Penfluridol inhibits Akt-GLI1 signaling when CHLA-200 cells were treated with different concentrations of penfluridol for 48 h. Representative blots showing concentration-dependent effect of penfluridol on pAkt(Ser473), Akt, GLI1, OCT4, Cl caspase 3 and Cl PARP. Actin was used as loading control. Figures shown are the representative blot of at least three independent experiments.

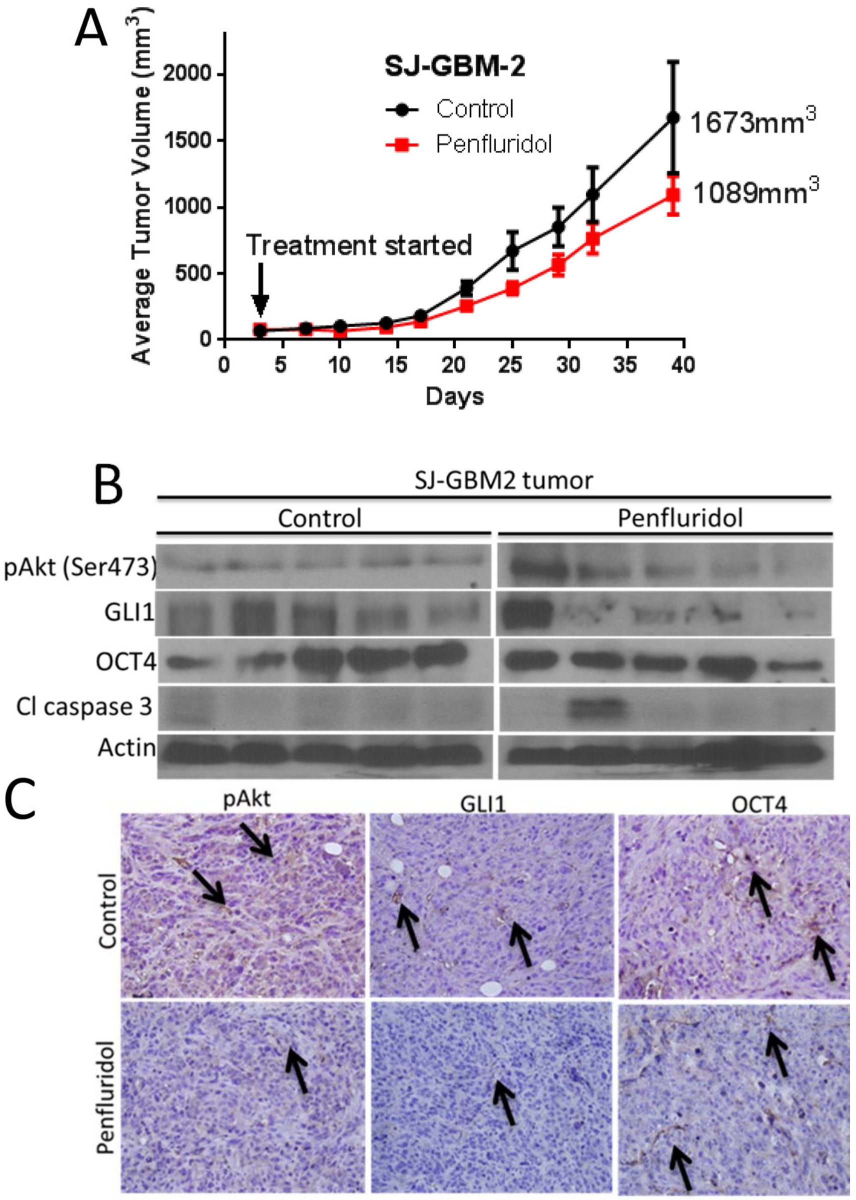

Supplementary Figure 2: Penfluridol suppresses the growth of pediatric-patient derived SJ-GBM2 tumors in xenograft model by inhibiting Akt-GLI1 signaling (**A**) 1×10^6 SJ-GBM2 cells in 1:1 mixture of PBS and matrigel were implanted in right and left flanks of 4-6 week old athymic nude mice ($n = 5$). Treatment with 10 mg/kg penfluridol by oral gavage everyday started 4 days after tumor cells injection till day 39. Values were plotted as means ± SEM. Tumors were removed after terminating the experiments, homogenized, lysed, and analyzed for pAkt(Ser473), GLI1, OCT4 and Cl caspase3 by western blotting. Actin was used as loading control. (**B**) Each lane of blot represents tumor from individual mouse. (**C**) Tumors were sectioned and immunostained for pAKT, GLI1 and OCT4 as described in materials and methods.

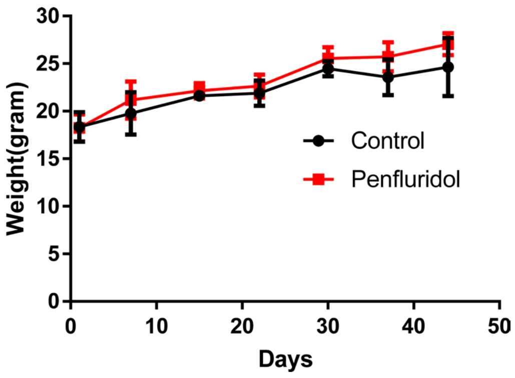

Supplementary Figure 3: Average weight of mice taken once in a week and plotted against days.

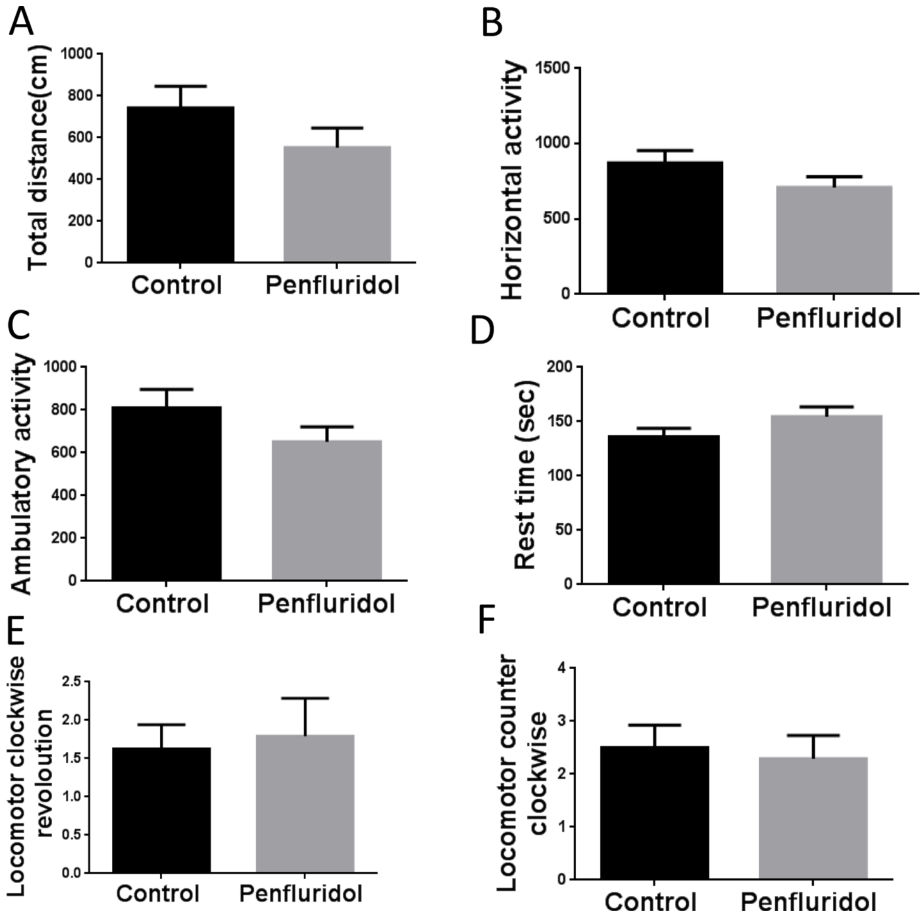

Supplementary Figure 4: Penfluridol does not affect any behavioral activity during the treatment of mice bearing brain tumors after chronic administration. In intracranial experiment, behavioral activity of mice was assessed using Versamax (Accuscan Instruments) after 54 days of 10 mg/kg penfluridol administration by oral gavage every day. (**A**) Total distance (**B**) Horizontal activity (**C**) Ambulatory activity (**D**) Rest time (**E**) Locomotor clockwise revolution (**F**) Locomotor counter clockwise revolution. Values were plotted as means ± SEM.
